# Effect of X-ray irradiation on the biological parameters of *Xestia c-nigrum*

**DOI:** 10.3389/fphys.2024.1362991

**Published:** 2024-02-21

**Authors:** Shijiao Chu, Bing Liu, Huan Li, Keke Lu, Yanhui Lu

**Affiliations:** ^1^ College of Agriculture, Shihezi University, Shihezi, China; ^2^ State Key Laboratory for Biology of Plant Diseases and Insect Pests, Institute of Plant Protection, Chinese Academy of Agricultural Sciences, Beijing, China; ^3^ Western Agricultural Research Center, Chinese Academy of Agricultural Sciences, Changji, China

**Keywords:** *Xestia c-nigrum*, sterile insect technique, x-ray irradiation, inherited infertility, longevity, survival

## Abstract

The sterile insect technique (SIT) is widely used to control Lepidopteran pests by inducing inherited sterility. The noctuid moth *Xestia c-nigrum* is a polyphagous pest whose subterranean larvae severely injure cereals and some vegetables. The goals of this study were to assess the impact of X-ray irradiation on the development and survival of *X. c-nigrum* and use the data to select suitable sterilizing doses for potential future use in pest management. Batches of male pupae were exposed to 0 (control), 10, 30, 50, 100, 200, 300, or 400 Gy of X-rays, approximately 24 h before adult emergence. Exposure of late-stage pupae to 10–200 Gy of radiation had no significant effect on adult emergence, but all doses (10–400 Gy) reduced adult longevity, the number of spermatophores in mated females, and the number of eggs laid per female in the irradiated parental generation compared with the controls. Exposure to 10 and 30 Gy had no significant effects in the F1 generation on 1) the rate of egg hatch, 2) the duration of larval or pupal development, or 3) adult longevity. However, exposure to 50 Gy reduced the rate of egg hatch in the F1 generation, and when male pupae were exposed to 100 Gy only 1% of the F1 eggs hatched. Also at 100 Gy, the developmental durations of larvae and pupae were significantly prolonged, and longevity of adult moths was reduced. There were no significant differences between the control group and any treatments in 1) the sex ratio of the F1 adults, 2) the duration of F1 pre-oviposition or oviposition periods, or 3) the number of eggs laid per F1 female. Our findings indicate that a dose of 100 Gy can effectively slow pest development and reduce larval survival in the F1 generation. In addition, F1 adults from lines treated with 100 Gy were able to mate and lay eggs, but all F2 eggs failed to hatch. Our results suggest that use of X-ray irradiation has potential to control this polyphagous pest at the regional level.

## Introduction

The spotted cutworm *Xestia c-nigrum* is an important agroforestry pest found in temperate and tropical regions of Asia, Europe, North Africa, and North America (Subchev et al., 1996). With a wide host range and high reproductive potential, it is an economically important pest (Broad and Boyes, 2022). Typical damage is to plant seedlings in the early spring season, when larvae feed on roots and sever stems at ground level, causing them to wilt and die (Wright et al., 2010). Due to the larvae’s nocturnal activity and underground feeding sites, their presence is often only revealed when the plants are already severely damaged, making them extremely difficult to control (Weng et al., 2019). Despite the use of light traps to monitor the flight period of the adults, it is still difficult to predict their damage (Hrudová et al., 2022). Chemical insecticides are still one of the most effective ways to suppress this pest; however, their widespread application can leave pesticide residues in crops, induce the emergence of highly resistant moths, and harm non-target organisms (Goudarzi et al., 2015).

The advantage of the sterile insect technique (SIT) is that it is specific to the target pest and its action is based on releasing males that confer sterility to females that the males seek out and mate with. This mode of action makes SIT particularly effective at controlling hidden pests, especially those whose larvae burrow or live in the soil (Shelly and Manoukis, 2022; Chen et al., 2023). For example, the release of sterile *Cydia pomonella* male moths in the Similkameen Valley of British Columbia in Canada from 1976 to 1978 resulted in 100% reduction of economic damage in orchards (Proverbs et al., 1982). In 1999, the release of sterile males of the tephritid fly *Ceratitis capitata* in the Hex River Valley in South Africa reduced wild fly populations 6 to 7-fold, greatly reducing crop losses due to this fruit fly (Barnes et al., 2004). SIT has been widely used in many countries against a variety of pests, including fruit flies [*C. capitata* (Plá et al., 2021), *Anastrepha ludens* (Ramírez-Santos et al., 2021), *Zeugodacus cucurbita* (Koyama et al., 2004)]; moths [*Agrotis ipsilon* (Salem et al., 2014), *Agrotis segetum* (Andreev and Martens, 1974), *C. pomonella* (Neven and Wakie, 2020)]; and beetles [*Anoplophora glabripennis* (IDIDS, 2003), *Callosobruchus analis* (Chiluwal et al., 2019)].

Historically, isotopes such as Cobalt 60 (^60^Co) and Caesium 137 (^137^Cs) that emit gamma radiation have been employed for sterilization (Klassen and Vreysen, 2021). In the late 1980s, preliminary studies investigated the effect of SIT on *X. c-nigrum* control through the application of gamma radiation, showing that a certain dosage of gamma rays could reduce the percentage of egg hatch in *X. c-nigrum* (Khattak et al., 1989). This study suggested that SIT might be a potential control method for this pest. More recently, studies on the effects of gamma radiation on insects have declined because gamma radiation sources are in need of regular replenishment, are very expensive, and are becoming more difficult to obtain due to stricter regulations (Mastrangelo et al., 2010; Gómez-Simuta et al., 2021). X-ray sources, in contrast, are increasingly employed in research applications due to their affordability, ease of transportation, and superior penetration capabilities compared to gamma rays. In addition, the irradiation process is simple to perform and lower risk (Kumano et al., 2018; Wang et al., 2023). However, the effect of X-ray irradiation on *X. c-nigrum* needs further assessment.

SIT is based on the sterilization of insects through radiation-induced mutations. When applying SIT, it is important to comprehensively consider the selection of the irradiation dose, appropriate release ratio, and dynamics of the target insect population in the release area to achieve the best effects (Judd et al., 2011; Orozco et al., 2013; Woods et al., 2016; Saour et al., 2022; Osouli et al., 2023). In particular, the efficiency of SIT is especially influenced by the correct choice of a suitable radiation dose (Ramírez-Santos et al., 2021). While high doses are obligatory for total sterility in Lepidoptera, such high doses can diminish adult longevity and mating competitiveness (Marec and Vreysen, 2019). In comparison, genetic sterility that manifests in the F1 offspring, which is induced through the use of lower doses of radiation, allows treated insects to retain relatively high mating competitiveness (Li, 2006). For example, Jiang et al. (2022a) found that X-rays applied at 25–150 Gy reduced the fertility of the F1 generation of *Spodoptera litura* without affecting mating ability. However, to determine the most effective sterilization dose for insects, it is important to evaluate several biological characteristics that may be affected by irradiation, including adult emergence rates, adult longevity, egg numbers from irradiated females, hatchability of eggs from such females, and the survival of the larval from these eggs (Zhao et al., 2022).

The aim of this research is to determine the dose of X-ray radiation that most effectively sterilizes pupae and the resulting adults of *X. c-nigrum*. This determination was made by measuring life history parameters, including for adults of the irradiated parental generation (1) adult emergence, longevity, the numbers of spermatophores produced, and numbers of eggs laid per female, as well as (2) in the F1 generation, the egg hatch rate, larval developmental duration, stage survival rates, pupal mass, adult longevity, sex ratio, and per female oviposition, and finally (3) for the F2 generation the hatch rate after X-ray radiation of pupae of the parental generation. These results will aid in the application of sterile insect techniques using X-ray radiation to manage *X. c-nigrum* by determining appropriate sterility parameters.

## Materials and methods

### Insects sources

In mid-July 2022, during the peak of the *X. c-nigrum* population in field, adults were collected by light-trapping at the Korla Experimental Stations of the Chinese Academy of Agricultural Sciences, Korla city, in southern Xinjiang Province, China. After being trapped, moths were kept in cages covered with gauze for egg laying and provided with a 20% honey solution daily as food. The colony was reared at 24°C ± 1 °C, 50% ± 10% RH, and a 12:12 (L:D) photoperiod. Eggs laid on the gauze were collected daily and held for hatching. Neonate larvae were inserted into glass tubes (2.5 cm dia, 7.5 cm h) and supplied with artificial diet (the main components being wheat germ, casein, agar, sucrose, Wesson’s salt mixture, and vitamin mixture, Zhang et al., 2012), with 5–10 larvae per tube. Fresh diet was provided every 2 days. After the larvae reached the fourth instar, they were placed individually in new tubes for pupation, and pupae were examined 10 days later to separate them by sex (Zhang et al., 2020). After egg hatch, larvae and pupae were reared under continuous dark 0:24 h (L:D).

Once this colony was well established, male pupae were collected for use in the irradiation test, isolating pupae approximately 24 h before adult emergence. Pupae used in our study originated from the second or third rearing generations.

### X-ray irradiation procedures

An X-ray irradiator (MultiRad 160, Faxitron Bioptics LLC, Tucson, AZ, United States of America) was used as the source of X-rays in our test. X-ray irradiation was done at ambient temperature, with the X-ray machine being operated at 160 kV and 25 mA, emitting 7.012 Gy/min at the point of irradiation. The machine and procedure were calibrated annually to ensure the accuracy of the dose administered and compliance with nationally recognized safety standards. The sample to be irradiated was placed in the center of the wire stage of the irradiator system, and X-rays are emitted from an X-ray tube positioned at a standard distance above the target. The irradiation voltage, current, and time were determined automatically based on the irradiation dose selected using the control panel. The irradiation doses used as treatments were 0 (no irradiation, i.e., the control), 10, 30, 50, 100, 200, 300, and 400 Gy. When the treatment’s dosage had been administered, X-ray emission automatically ended, and the irradiated insects were then removed and used for the lifetable experiment.

### Effect of irradiation on insect emergence, longevity, the per female oviposition rate, and the number of spermatophores in mated female in the parental generation

Groups of male pupae were placed in Petri dishes (90 mm dia) 48 h before adult emergence and irradiated with 10, 30, 50, 100, 200, 300, or 400 Gy, together with an untreated control. At least 50 pupae were treated in each group. Irradiation was done in four batches, each including all treatment doses and a control. The irradiated pupae were then transferred to rearing boxes and held until adults emerged. Pupae from which no adults emerged were counted to evaluate emergence rates. Emerged adult males were paired with non-irradiated, virgin females in disposable transparent plastic cups (8 cm dia at the mouth, 5 cm dia at the bottom, and 13 cm h) for mating. Paired moths were supplied with a 20% honey solution.

To determine the effect of treatment on moth oviposition, we evaluated four replicates of 30 pairs of adults for each treatment, including the controls. The mouth of each cup was covered with gauze for oviposition and replaced daily to record egg production. If male moths died, one male moth from the same treatment was added to continue mating with the paired female moths until the female moth died. The boxes with moths were kept under the same conditions as the rearing colony until all adults died. Longevity of the male adults was determined by recording daily mortality in each box.

To determine the effect of treatment on male sperm production and transfer, adults from irradiated male pupae were paired with newly emerged, non-irradiated virgin females. At various time points after pairing (1, 2, 3, 4, 5, and 10 days), females were sacrificed and dissected in a 0.8% NaCl solution, and the number of spermatophores in each female’s bursa copulatrix was determined using a stereomicroscope. There were four replicates, each of five pairs, for each treatment, including the controls, and each date of female dissection, for a total of 960 pairs (8 treatments or control x 4 replicates x 5 pairs x 6 time points for dissection).

### Effect of irradiation on development and fecundity of the F1 generation

To determine the effect of mating with irradiated males on the fertility of F1 eggs from non-irradiated females, batches of eggs laid 3–5 days after pairing in each treatment were collected to record their hatching rate. There were 300 eggs in each replicate of each treatment, and there were four replicates. Eggs were observed daily to detect hatch, until all the eggs had either hatched or died and dried up. The number of eggs that did not hatch was counted and used as the measure of the level of sterility induced by the irradiation treatments.

After viable eggs from each of the above treatments had hatched, the neonate larvae (within 12 h of hatching) were transferred into glass tubes containing artificial diet and reared until pupation. Larvae that did not pupate normally were scored to measure the survival rate of larvae. Pupal weights were measured 7 days after pupation, for both pupae from which adults emerged and that had died as pupae. The pupal duration and emergence rates for each treatment were determined. There were four replicates, each with 100 individuals, for each treatment, including the controls. The sex of each emerged adult was determined, and the males were then paired with non-irradiated females. Females were not used for further tests. Longevity of adults of both sexes was recorded until all adults had died.

### Effect of irradiation on egg hatch rate of F2 generation

To determine the intergenerational effect of irradiated grand-parents, F1 males sired by irradiated parental males were paired with unirradiated females. Eggs laid 3–5 days after this pairing were observed for hatching and the number of eggs that did not hatch was taken as the rate of induced sterility in the F2 generation.

### Statistical analysis

The eclosion rate of pupae of the irradiated parental generation, and the survival rate of larvae of the F1 generation were all analyzed using generalized linear models (GLM) with binomial distributions. The number of spermatophores in mated females of the parental generation were analyzed using a GLM with a Poisson distribution. Data on adult longevity of the parental generation, the F1 larval and pupal developmental times, adult longevities of F1 males and females (and pooled), F1 pupal weight, sex-ratio (calculated as the proportion of female moths: F/(F + M)), pre-oviposition and oviposition periods, F1 oviposition number, and both F1 and F2 egg hatching rate were analyzed using one-way ANOVAs. Oviposition levels of females of the parental generation were analyzed using a GLM with negative binomial distributions, which was done using the “glm.nb” function within “MASS” package (Venables and Ripley, 2002). Within each GLM or ANOVA analysis, each parameter in the life tables of the F1 generation were compared between the various radiation doses and the control using Dunnett test. For other analyses, multiple comparisons were made using Tukey’s Honestly Significant Difference (HSD) correction (for *p* < 0.05). Analysis of the survival from first instars to the adult stage for the F1 generation was done using Cox Regression by the “survifit” function within the “survival” package (Therneau, 2023). All analyses were performed by using R 4.3.1 software (R Development Core Team, 2023).

## Results

### Effect of irradiation on insect emergence, longevity, the per female oviposition, and the number of spermatophores in mated female in the parental generation

**FIGURE 1:**
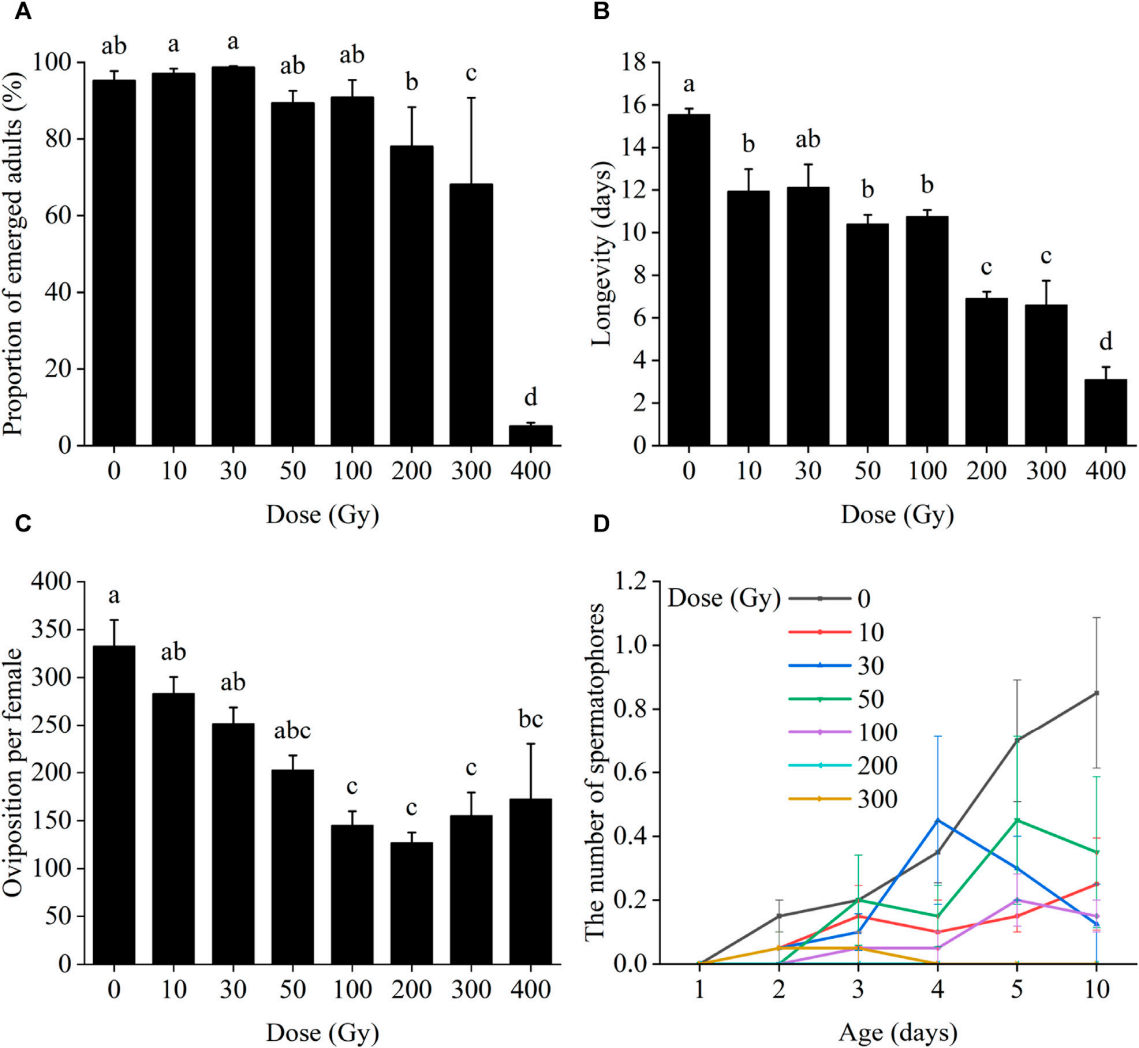
Effects of pupal irradiation on **(A)** the proportion of successfully emerged parental adults, **(B)** longevity of adults of the parental generation, **(C)** oviposition per parental female and **(D)** the number of spermatophores of mated females of the parental generation, with different doses. Data are average ±SE; means followed by the same lowercase letters are not statistically different (*p* > 0.05, Tukey HSD).

Irradiation of male pupae at doses ≥300 Gy significantly reduced adult emergence compared to the control group (0 Gy) (Figure 1A). Only 68.2% and 5.1% of irradiated male pupae reached the adult stage at 300 and 400 Gy, which was a significant difference between those doses (*χ* ^2^ = 436.92, *df* = 7, *p* < 0.001). Male pupal emergence at doses from 10 to 200 Gy, however, did not differ significantly.

Longevity of the male adults of the parental generation decreased progressively with increased radiation doses, changing from 12.13 to 3.11 days from 10 to 400 Gy (Figure 1B). Adult longevity at all doses differed from that of the controls except for 30 Gy (*F*_7,24_ = 28.16, *p* < 0.001), at which dose adult longevity was not significantly different from the control group (12.13 days vs. 15.55).

When male pupae were exposed to radiation and mated with unexposed females, the radiation dosage did not affect the number of eggs laid per female of the same generation until male moths were exposed to 100 Gy or more of radiation (*χ* ^2^ = 59.58, *df* = 7, *p* < 0.001; Figure 1C).

The number of successful matings between irradiated males and unirradiated females ranged from 0 to 4, all of which occurred after day 2 (after pairing). But there were no significant differences between those doses on day 2 (*χ* ^2^ = 1.69, *df* = 6, *p* = 0.946), day 3 (*χ* ^2^ = 1.74, *df* = 6, *p* = 0.942), day 4 (χ^2^ = 5.15, *df* = 6, *p* = 0.524), day 5 (*χ* ^2^ = 6.94, *df* = 6, *p* = 0.326) or day 10 (*χ* ^2^ = 8.16, *df* = 6, *p* = 0.227) (Figure 1D).

### Effect of irradiation on development and fecundity of the F1 generation

**FIGURE 2:**
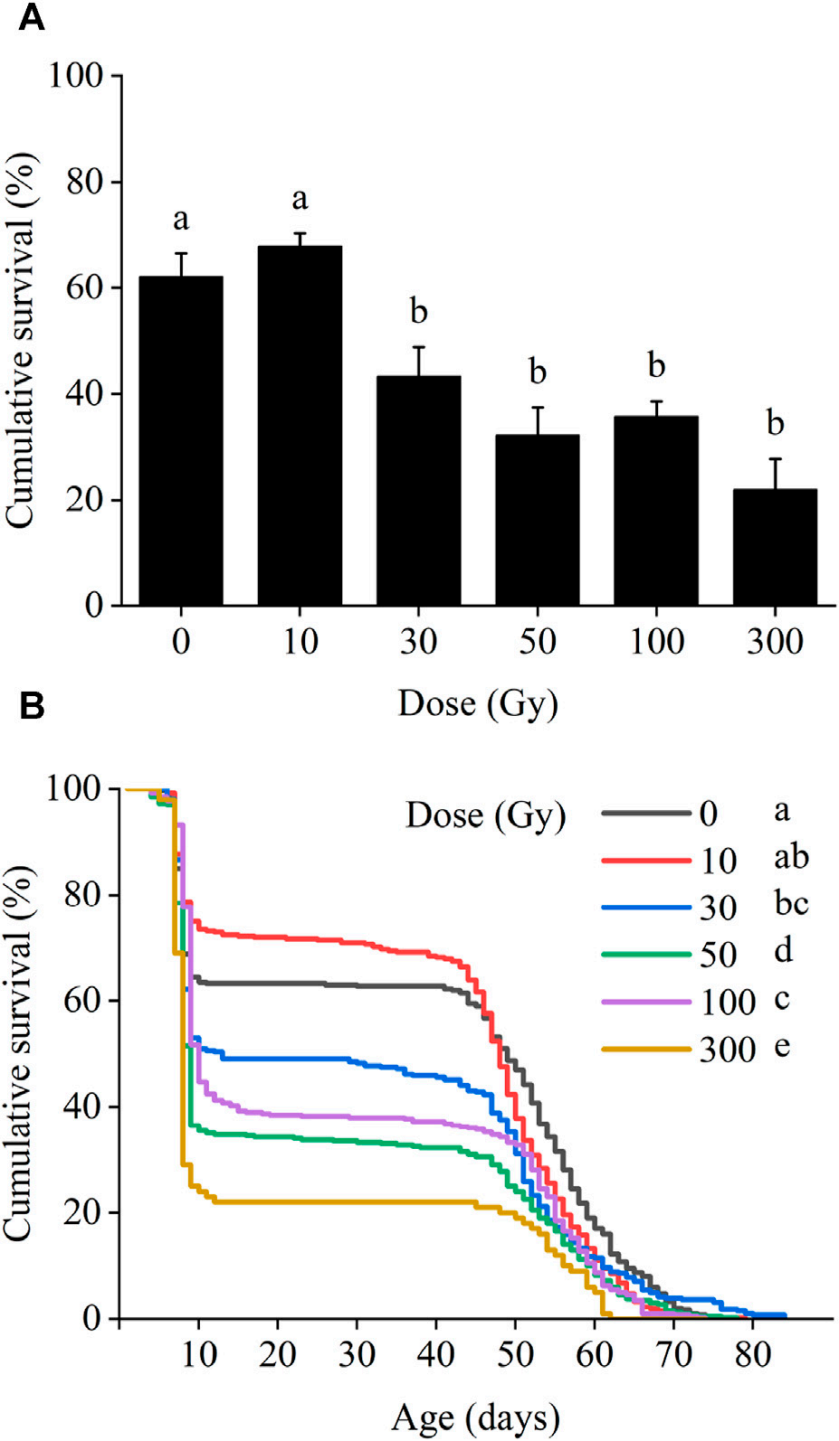
Effects of pupal irradiation on the survival rate of **(A)** larvae and **(B)** from first instar larvae to adult death of the F1 generation, with different doses. Data are average ±SE; means followed by the same lowercase letters are not statistically different (*p* > 0.05, Tukey HSD).

In the F1 generation, larval survival was significantly affected by the irradiation dose, with larval survival gradually decreasing as the dose of radiation increased (*χ* ^2^ = 68.85, *df* = 5, *p* < 0.001; Figure 2A). Larval survival rates at 10 and 30 Gy were 67.9% and 43.3%, respectively. With increasing irradiation, larval survival declined to 32.3%, 35.8%, and 22.0% following exposure to 50, 100, and 300 Gy, respectively, all of which were statistically different from the control level (62.3%).

Survival from egg hatch was reduced at all radiation doses above 30 Gy (*χ* ^2^ = 79.15, *df* = 5, *p* < 0.001). Initially, population survival of the first larval instar was high in all treatments for the first 7 days. However, between 7 and 10 days period from first to second instar larvae, cumulative survival decreased sharply in all treatments including the control and continued to decrease steadily between 10 and 50 days. After 50 days, cumulative survival again declined more sharply until adult death (Figure 2B).

The developmental parameters (see Table 1) of F1 larvae, pupae, and adults following X-ray irradiation of the parental male pupae showed that egg hatch was reduced by increased doses of radiation. At 10 and 30 Gy, egg hatch was 58.9% and 41.3%, respectively, and these values were not significantly different from the control (51.9%); but all doses ≥50 Gy yielded egg hatch rates significantly lower than the control, reaching 0% at 200 and 400 Gy (*F*_5,18_ = 10.17, *p* < 0.001). Different doses of irradiation had significant effects on the duration of all larval instars, including first: *F*_5,18_ = 9.79, *p* < 0.001, second: *F*_5,18_ = 15.14, *p* < 0.001, third: *F*_5,18_ = 5.91, *p* = 0.002, fourth: *F*_5,18_ = 4.06, *p* = 0.012, fifth: *F*_5,18_ = 10.67, *p* < 0.001, sixth: *F*_5,18_ = 7.62, *p* < 0.001, and the whole larval stage (*F*_5,18_ = 9.36, *p* < 0.001), as well as the pupal stage (*F*_5,18_ = 5.46, *p* = 0.003), the first instar larval-pupal stage (*F*_5,18_ = 9.79, *p* < 0.001), and pupal weight (*F*_5,18_ = 11.18, *p* < 0.001) in the F1 generation.

**TABLE 1:** Effect of irradiation (Gy units) on developmental duration and fecundity of F1 generation.

Stage	Parameters	0 (Control)	10	30	50	100	300	*Df*	*F*	*p*
Egg	hatch rate (%)	51.92 ± 8.36	58.85 ± 8.71	41.33 ± 9.06	20.89 ± 4.48 *	1.08 ± 0.77 ***	11.21 ± 8.44 **	5	10.168	<0.001
Larvae	1st instar duration (d)	6.26 ± 0.10	5.74 ± 0.09	6.02 ± 0.18	6.59 ± 0.11	6.92 ± 0.06 *	6.53 ± 0.21	5	9.791	<0.001
	2nd instar duration (d)	3.71 ± 0.10	3.41 ± 0.03	3.65 ± 0.06	4.02 ± 0.06	4.36 ± 0.07 ***	3.90 ± 0.14	5	15.135	<0.001
	3rd instar duration (d)	3.31 ± 0.05	3.27 ± 0.07	3.39 ± 0.18	3.46 ± 0.17	3.95 ± 0.06 *	3.99 ± 0.18 **	5	5.909	0.002
	4th instar duration (d)	3.52 ± 0.07	3.61 ± 0.13	4.11 ± 0.35	3.77 ± 0.08	4.00 ± 0.12	4.53 ± 0.21 **	5	4.057	0.012
	5th instar duration (d)	5.31 ± 0.25	5.78 ± 0.26	5.89 ± 0.35	5.07 ± 0.09	5.28 ± 0.15	7.42 ± 0.36 ***	5	10.606	<0.001
	6th instar duration (d)	7.17 ± 0.24	7.22 ± 0.14	7.88 ± 0.48	8.15 ± 0.16	8.99 ± 0.37 **	9.00 ± 0.20 **	5	7.621	<0.001
	larvae stage duration (d)	26.82 ± 0.64	26.74 ± 0.28	28.63 ± 1.21	29.25 ± 0.35	32.44 ± 0.68 ***	29.63 ± 0.55 *	5	9.356	<0.001
Pupa	Duration (d)	14.22 ± 0.28	14.40 ± 0.12	14.02 ± 0.14	14.53 ± 0.30	15.33 ± 0.09 **	14.53 ± 0.09	5	5.463	0.003
	Mass (mg)	286.96 ± 6.93	253.40 ± 5.82 **	248.22 ± 5.05 ***	294.10 ± 5.20	271.06 ± 5.53	271.55 ± 2.95	5	11.176	<0.001
1st instar larval-pupal duration (d)		41.04 ± 0.85	41.14 ± 0.33	42.00 ± 0.64	43.65 ± 0.64 *	46.39 ± 0.51 ***	43.93 ± 0.82 *	5	9.786	<0.001
Adult	Female longevity (d)	18.48 ± 1.32	14.20 ± 1.07	16.17 ± 0.40	15.12 ± 1.46	12.60 ± 1.37	13.71 ± 3.07	5	1.571	0.219
	Male longevity (d)	10.20 ± 0.54	8.80 ± 0.27	9.09 ± 0.47	9.24 ± 1.20	8.06 ± 0.26	9.04 ± 1.43	5	0.694	0.635
	All adult longevity (d)	14.20 ± 0.86	11.55 ± 0.60	12.19 ± 0.38	11.98 ± 0.90	9.74 ± 0.96 *	11.52 ± 1.66	5	2.171	0.103
	sex ratio (F/(F + M))	0.48 ± 0.05	0.50 ± 0.05	0.44 ± 0.05	0.44 ± 0.03	0.36 ± 0.07	0.48 ± 0.07	5	0.733	0.608
	Pre-oviposition period (d)	5.08 ± 0.79	2.90 ± 0.43	2.53 ± 0.39 *	2.56 ± 0.53	2.91 ± 0.17	2.71 ± 1.13	5	2.247	0.009
	Oviposition period (d)	11.12 ± 0.56	9.75 ± 1.07	11.85 ± 0.49	10.34 ± 1.81	6.93 ± 1.66	10.25 ± 3.27	5	0.934	0.482
	Egg deposited per female	332.34 ± 19.64	261.88 ± 29.89	318.38 ± 52.81	257.42 ± 33.49	164.23 ± 11.67	285.71 ± 127.82	5	0.991	0.451

Data are shown as average ±SE; *df*, *F*, and *p* values are the results after one-way ANOVA, and asterisks in the table indicate significant differences between the treatment groups and the control; *p < 0.05, **p < 0.01, ***p < 0.001 (Dunnett’s multiple comparisons).

In contrast, longevity and sex ratio were not affected by irradiation: (1) male longevity (*F*_5,18_ = 0.69, *p* = 0.635), (2) female longevity (*F*_5,18_ = 1.57, *p* = 0.219), (3) the longevity of all adults combined (*F*_5,18_ = 2.17, *p* = 0.103), (4) sex ratio (*F*_5,18_ = 0.73, *p* = 0.608). The pre-oviposition period (*F*_5,18_ = 2.25, *p* = 0.009), and oviposition period (*F*_5,18_ = 0.93, *p* = 0.482), and oviposition per female (*F*_5,18_ = 0.99, *p* = 0.451) at all irradiation doses were not significantly different from those of the controls. The results indicate that, in the F1 generation, irradiation of the previous generation’s males mainly affected the larvae of the F1 generation.

As for the control moths, the sex ratio (F/(F + M) of irradiated moths was male biased = 0.44–0.36) in the 30–100 Gy irradiation groups. F1 adult longevity did not show significant differences between treatment groups in one-way ANOVA (*p* = 0.103). However, Dunnett’s method (which was used to compare with the difference between each treatment group and the control group) revealed that the adult longevity at 100 Gy (9.74 days) was significantly lower than that of the control group, which was 14.20 days (*p* = 0.019; Table 1).

### Effect of irradiation on egg hatch of F2 generation

**FIGURE 3:**
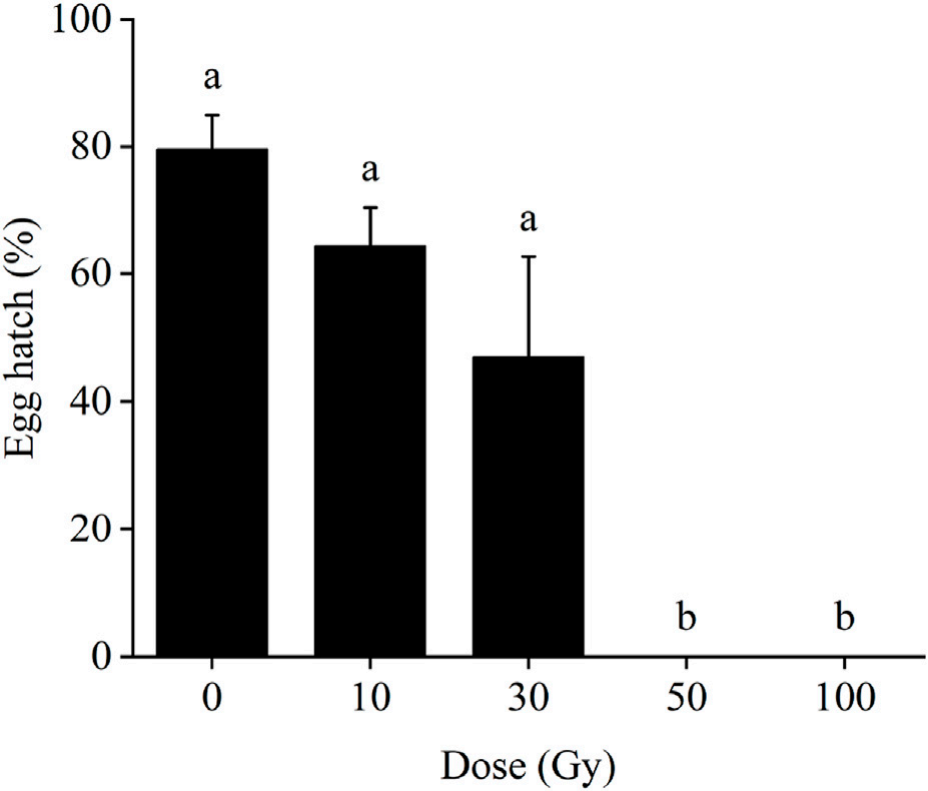
Effect of pupal irradiation on egg hatch rate of the F2 generation. Data are average ±SE; means followed by the same lowercase letters are not statistically different (*p* > 0.05, Tukey HSD).

In the F2 generation, egg hatch was significantly reduced as the dose of radiation applied increased (*F*_4,15_ = 21.75, *p* < 0.001; Figure 3). In the control group, the egg hatch rate was 79.6%, but this decreased to 64.5% and 47.0% at 10 and 30 Gy, respectively. These values, however, were not significantly different from the control. However, at 50 Gy and 100 Gy, the rate was reduced to 0%, showing that at these doses no eggs of the F2 generation survived.

## Discussion

The objective of SIT is to prevent the reproduction of females in invasive or established pest populations through the distribution of mass-produced, sterile insects, with particular emphasis on sterile males (Light et al., 2015; Klassen and Vreysen, 2021). The success of SIT is based on the use of a dosage of radiation that sterilizes male insects, but still leaves them as competitive as their fertile counterparts within the target population (M'Saad Guerfali et al., 2011; Pérez-Staples et al., 2012; Kapranas et al., 2022). Sterility can manifest at various life stages of the targeted insect during its reproductive development, such as failure of egg production or hatch, larval mortality, failure to pupate, non-emergence of adults, and sterility of any F1 adults that do emerge (Pérez-Staples et al., 2012; Light et al., 2015; Kapranas et al., 2022).

Following pupal irradiation, rates of adult emergence from irradiated pupae and adult longevity are important measures of the quality of the sterile males being produced (Mastrangelo et al., 2010). Reduction in the emergence rate of male adults after irradiation is a dose responsive outcome, with emergence decreasing as dosage increases. This relationship implies that higher radiation doses can induce morphological damage in male adults, contrary to the goal of SIT (Aksoy et al., 2017). We found that the emergence rate of male pupae of *X. c-nigrum* was not affected by dosage within the range of 10–200 Gy. However, the emergence rate was significantly depressed if the radiation dose was 300 Gy or higher. Also, our results showed that an increase in radiation exposure in male pupae lead to a significant reduction in adult longevity, suggesting that exposure to high levels of radiation may also affect the somatic cells of insects (Brower, 1976).

The number of spermatophores present in a female moth’s bursa copulatrix is an indicator of her number of successful matings, with each spermatophore originating uniquely from one male moth (Wu, 2020). In this study, we found that the number of spermatophores transferred to females by irradiated males declined gradually with increasing irradiation dose for each day of pairing. Moreover, mating virtually ceased at radiation doses exceeding 200 Gy. Meanwhile, the number of spermatophores in the bursa copulatrix of females increased with days of pairing, as seen in a study of *Anopheles arabiensis* (Helinski and Knols, 2009).

Ideally in SIT, an optimum amount of irradiation is used so that the sterility level of treated insects increases and the number of eggs and their hatch rate decreases (Mansour and Mohamad, 2004). In our study, the number of eggs laid by the parental generation was significantly reduced by the radiation treatments, and the rate of egg hatch in the F1 generation was significantly reduced compared to the control at ≥ 100 Gy. These results suggest that X-ray irradiation may damage the reproductive cells and ultimately cause sterility of this pest. Similar findings have been reported for *Drosophila melanogaster* and *Sesamia nonagrioides* (Vaiserman et al., 2004; Aksoy et al., 2017). In contrast, irradiation of *C. pomonella* at 366 Gy with X-rays did not have any impact on egg numbers but significantly reduced egg viability (Zhang et al., 2023). These variations suggest that the effects of irradiation depend on the target species.

Some studies have reported that use of X-rays for irradiation of pupae caused a dose-dependent reduction in larval survival, adult emergence, and, in the next-generation, of fecundity and F1 adult longevity (Kim et al., 2015; Jiang et al., 2022a; Jiang et al., 2022b). In our study, as the irradiation dose increased, the development rates decreased for both larvae and pupae of the F1 generation after irradiation, while the longevity of the adults, the duration of pre-oviposition period and the whole oviposition periods were shortened. Also, there was a reduction in the survival rate of both larvae and the whole period from first instar larvae to adult. In addition, the sex ratio of adults of the F1 generation of moths following the irradiation of their male parent became more male biased as the dose increased. This finding is consistent with research on *Phyllocnistis citrella* using gamma rays at 100–250 Gy and on *Ostrinia nubilalis* using 90–180 Gy (Osouli et al., 2023). Moreover, oviposition per female in the F1 generation declined progressively with increasing irradiation dose, and at the 50 and 100 Gy levels, there was a complete loss of egg hatch in the F2 generation.

This study examined the physiological responses of *X. c-nigrum* to X-ray irradiation, including development and reproduction. However, before these findings can be applied at a field scale, the post-treatment effects of irradiation on moth behaviors such as on flight ability and mating competitiveness, as well as the impact of different ratios of irradiated males to wild moths, need to be examined. Preliminary findings from this study suggest that employing 100 Gy of X-rays irradiation may be effect for SIT programs against *X. c-nigrum*.

## Data Availability

The raw data supporting the conclusion of this article will be made available by the authors, without undue reservation.
